# Identification of the cellular components involved in de novo immune hepatitis: a quantitative immunohistochemical analysis

**DOI:** 10.1186/s12967-018-1440-8

**Published:** 2018-03-13

**Authors:** Elena Aguado-Domínguez, Lourdes Gómez, José Manuel Sousa, Miguel Ángel Gómez-Bravo, Antonio Núñez-Roldán, Isabel Aguilera

**Affiliations:** 1 Department of Immunology, Instituto de Biomedicina de Sevilla (IBiS), Hospital Universitario Virgen del Rocío/CSIC/University of Seville, Avda. Manuel Siurot s/n, 41013 Seville, Spain; 2 Department of Pathology, Instituto de Biomedicina de Sevilla (IBiS), Hospital Universitario Virgen del Rocío/CSIC/University of Seville, Seville, Spain; 3 Department of Digestive Diseases, Instituto de Biomedicina de Sevilla (IBiS), Hospital Universitario Virgen del Rocío/CSIC/University of Seville, Seville, Spain; 4 Liver Transplant Unit, Instituto de Biomedicina de Sevilla (IBiS), Hospital Universitario Virgen del Rocío/CSIC/University of Seville, Seville, Spain

**Keywords:** Inflammatory infiltrates, Liver biopsy, De novo immune hepatitis, newCAST, Chronic rejection, Donor/recipient mismatch, GSTT1

## Abstract

**Background:**

Diagnosis of de novo immune hepatitis (dnIH) after liver transplantation relies on biopsy findings, with an abundance of plasma cells (PCs) in the inflammatory infiltrates a hallmark of the disease. Very little is known about what other types of immune cells exist in the infiltrates mainly located in the portal areas of the liver tissue.

**Methods:**

We analyzed the composition of T cells, B cells, PCs, and macrophages in the liver biopsies of 12 patients with dnIH, 9 of them obtained at the time of diagnosis. For comparison, biopsies from 9 patients with chronic rejection (CR) were included in the study. The results were analyzed by a computer-assisted stereology quantification method.

**Results:**

The major components of the infiltrates in the portal areas were CD3^+^ T lymphocytes in both groups, with 36.6% in the dnIH group versus 49.4% in the CR group. CD20^+^ B lymphocytes represented 14.9% in the dnIH group and 29.1% in the CR group. Macrophage levels were very similar in the dnIH and CR group (19.7% versus 16.8%, respectively). PCs were much less represented in CR biopsies than those from the dnIH group (mean value of 4.7% versus 28.8%).

**Conclusion:**

In conclusion, the determination of a characteristic cellular profile could be an important tool for a more reliable diagnosis of dnIH, in support of the histological evaluation made by the pathologist, which in most cases is challenging. Recognition of this condition is crucial because it leads to graft failure if left untreated.

**Electronic supplementary material:**

The online version of this article (10.1186/s12967-018-1440-8) contains supplementary material, which is available to authorized users.

## Background

De novo immune hepatitis (dnIH) is a dysfunction of the liver allograft that resembles native liver autoimmune hepatitis (AIH). Over the years, the nature of this response has been debated, although more recently, several reports sustained that de novo AIH was a variant of rejection and not a true autoimmune process [1–3]. In the group of patients diagnosed with dnIH in our hospital, we described the enzyme glutathione S-transferase T1 (GSTT1) as the target antigen of the immune response when a patient homozygous for the deletion allele (GSTT1*0/0), receives a liver from a donor with one or two functional copies of the gene (GSTT1*A/0, GSTT1*A/A) [4]. As a result, this drug metabolizing enzyme, highly active in the liver and expressed only in the graft, is recognized as a foreign antigen. In our experience, the rejection occurs within 2 years of the liver transplant, is always preceded by production of anti-GSTT1 antibodies, and is associated with receiving cyclosporine as the main initial immunosuppressor. In contrast, the use of tacrolimus as a calcineurin inhibitor has a protective effect preventing anti-GSTT1 antibody production and subsequently the development of dnIH [5]. The main subclasses of anti-GSTT1 antibodies have been characterized as IgG1 and IgG4 at similar proportions [6].

Distinguishing dnIH by liver biopsy is challenging because it shares some histological and clinical features with late onset acute rejection or with other post-transplant pathologies such as recurrent hepatitis C virus [7]. Apart from the presence of plasma cell (PC)-rich infiltrates in portal tracts, histological features may include portal bridging fibrosis, interface hepatitis, centrilobular hepatocyte necrosis or rosette formation. As biopsy findings are variable and not specific, diagnosis of dnIH cannot be made or excluded by histology alone. In the last update of the Banff Working Group on liver allograft pathology, the experts agreed on the need to replace the old terminology with the more appropriate “plasma cell-rich rejection”, in which antibodies to GSTT1 and HLA donor-specific antibodies are contributory but nonessential features [8].

In this study, we quantified the main cellular types of inflammatory infiltrates in the portal tracts of diagnostic biopsies of patients with dnIH. A group of liver transplanted patients with chronic rejection (CR) was also studied. To date, very few studies have investigated the composition of the cellular infiltrates and the relative contribution of the cell types involved in dnIH aside from the recognition of a PC population. An accurate, unbiased, and reproducible tool has been designed by our group in order to reduce intra- and inter-observer variability as well as to provide a precise cell representation in the tissue.

The aim of this study was to identify the cell types relevant for the immune response after liver transplantation from diagnostic biopsies of patients with dnIH, and to establish a common cellular profile (if there is one) using a novel protocol based on computer-assisted stereology quantification.

## Methods

### Patients

A cohort of 12 liver transplanted patients with a diagnosis of dnIH was included in the study. Liver transplantations were performed between 1996 and 2007 at Hospital Universitario Virgen del Rocío, Seville, Spain. Biopsies were harvested under clinical indication, from 1997 to 2012. No biopsy was collected solely for the purpose of this study. Two transplant recipients (patients 3 and 4) had a second follow-up biopsy collected months after diagnosis, and one patient (patient 7) had a biopsy obtained 4 months prior to diagnosis. A total of 15 specimens were analyzed. All cases were clinically and histologically reviewed in order to confirm dnIH diagnosis by physicians and pathologists. Eleven of the 12 patients received cyclosporine as baseline immunosuppression versus one patient who received tacrolimus. Nine additional biopsies from 9 patients who experienced CR were also included as a control group, where 7 of them had tacrolimus-based immunosuppression (Table 1).

**Table 1:** Characteristics of the patients with de novo immune hepatitis and chronic rejection

Patient	Gender	Original disease	Baseline IS	Biopsy date (biopsy #)	Months after LT	Diagnosis
1	F	Drug toxicity	CYA, AZA, ST	18-02-00^a^	21	dnIH
2	M	Alcoholic cirrhosis	CYA(N), MMF, ST	15-03-04^a^	58	dnIH
3	M	HCV + alcoholic cirrhosis	CYA(N), ST, anti-CD25	13-07-04 (B1)^a^ 05-09-04 (B2)	32 34	dnIH
4	F	Alcoholic cirrhosis	CYA, MMF, ST	16-07-04 (B1)^a^ 13-06-05 (B2)	10 21	dnIH
5	F	Unknown	CYA, MMF, ST	28-01-10^a^	32	dnIH
6	F	PBC	CYA, MMF, ST	19-04-10^a^	13	dnIH
7	F	HCV cirrhosis	CYA, MMF, ST	30-07-12 (B1) 05-12-12 (B2)^a^	3 8	HCV + AR dnIH
8	M	HCV cirrhosis	TAC, ST	28-12-00^a^	14	dnIH
9	F	HCV cirrhosis	CYA, MMF, ST	18-06-09^a^	57	dnIH
10	F	SBC	CYA (N), MMF, ST	19-02-14^a^	87	dnIH
11	F	HCV cirrhosis	CYA, AZA, ST	25-04-97^a^	7	dnIH
12	F	HCV cirrhosis	CYA(N), ST, anti-CD25	04-02-02^a^	19	dnIH
CR-1	F	HCV cirrhosis	TAC, MMF, ST	09-11-04	29	CR
CR-2	F	PBC	Sotrastaurin	01-11-11	12	Early CR
CR-3	M	Caroli disease	TAC, MMF, ST	08-03-13	17	CR
CR-4	M	Alcoholic cirrhosis + NASH	TAC, MMF, ST, anti-CD25	30-07-13	6	Early CR
CR-5	M	HCV cirrhosis + HC	CYA, MMF, ST	07-06-13	11	CR
CR-6	M	Cryptogenic	TAC, MMF, ST	08-03-12	9	Early CR
CR-7	M	Re-transplant	TAC, MMF, ST	05-08-10	74	CR
CR-8	M	Alcoholic cirrhosis	TAC, anti-CD25	12-03-14	84	CR
CR-9	M	PBC	TAC, anti-CD25	22-01-07	75	CR

M, male; F, female; LT, liver transplant; HCV, hepatitis C virus; PBC, primary biliary cirrhosis; SBC, secondary biliary cirrhosis; IS, immunosuppression; TAC, tacrolimus; AR, acute rejection; CYA, cyclosporine; N, Neoral; MMF, mofetil mycophenolate; ST, steroids; AZA, azathioprine; dnIH, de novo immune hepatitis; CR, chronic rejection; NASH, non-alcoholic steatohepatitis; HC, hepatocellular carcinoma

^a^dnIH diagnostic biopsy

All specimens were anonymized and were handled according to the ethical guidelines of the current Declaration of Helsinki, with the approval of the Hospital Universitario Virgen del Rocío ethical committee.

### Cell markers

Mouse monoclonal anti-human CD20 (clone L26), rabbit polyclonal anti-human CD3, and mouse monoclonal anti-human CD68 (clone PG-M1) were ready to use, and mouse monoclonal anti-human CD138 (clone MI15) was used at 1:80 dilution, all of them from Dako (Agilent Technologies, Santa Clara, California, United States of America); mouse monoclonal anti-human IgG4 (HP6025, Thermo Scientific, Rockford, Illinois, United States of America) was used at 1:500 dilution.

### Immunohistochemistry

Three-micrometer-thick serial sections of formalin-fixed, paraffin-embedded tissue sections were dewaxed in xylene, rehydrated in a series of ethanol, and heat-treated in citrate buffer (Dako, target retrieval solution, ref. S2031) for antigenic unmasking for 20 min. Sections were blocked for 40 min using blocking reagent (Roche Diagnostics, Mannheim, Germany) in maleic acid. Primary antibodies were incubated for 60 min at room temperature using a humid chamber. EnVision™ mixed secondary antibody (Dako, ref. K8000) was incubated for 40 min at room temperature using a humid chamber after several washes in PBS. The immunoreaction was completed with 3,3′-diaminobenzidine (DAB). Tissue sections were counterstained with hematoxylin. The same protocol was followed but omitting the primary antibody as negative control.

### Areas of inflammation

Immunostained slides were examined by a pathologist, blinded to diagnosis details. An exhaustive stereology evaluation was also performed, avoiding observer subjectivity, using new Computer-Assisted System Technology (newCAST™) by Visiopharm^®^ (Visiopharm, Hoersholm, Denmark) equipped with an Olympus BX61 (Olympus, Volketswil, Switzerland).

The area of portal inflammation was measured and cells were quantified as described below. GraphPad Prism^®^ Software (La Jolla, California, United States of America) was used for results representation, presented as mean and standard deviation for continuous variables and percentages. The results were considered statistically significant when p values were < 0.05.

### Cell quantification

Every tissue section corresponding to one cell marker was analyzed using the meander sample tool of the newCAST™ stereology system.

**Fig. 1:**
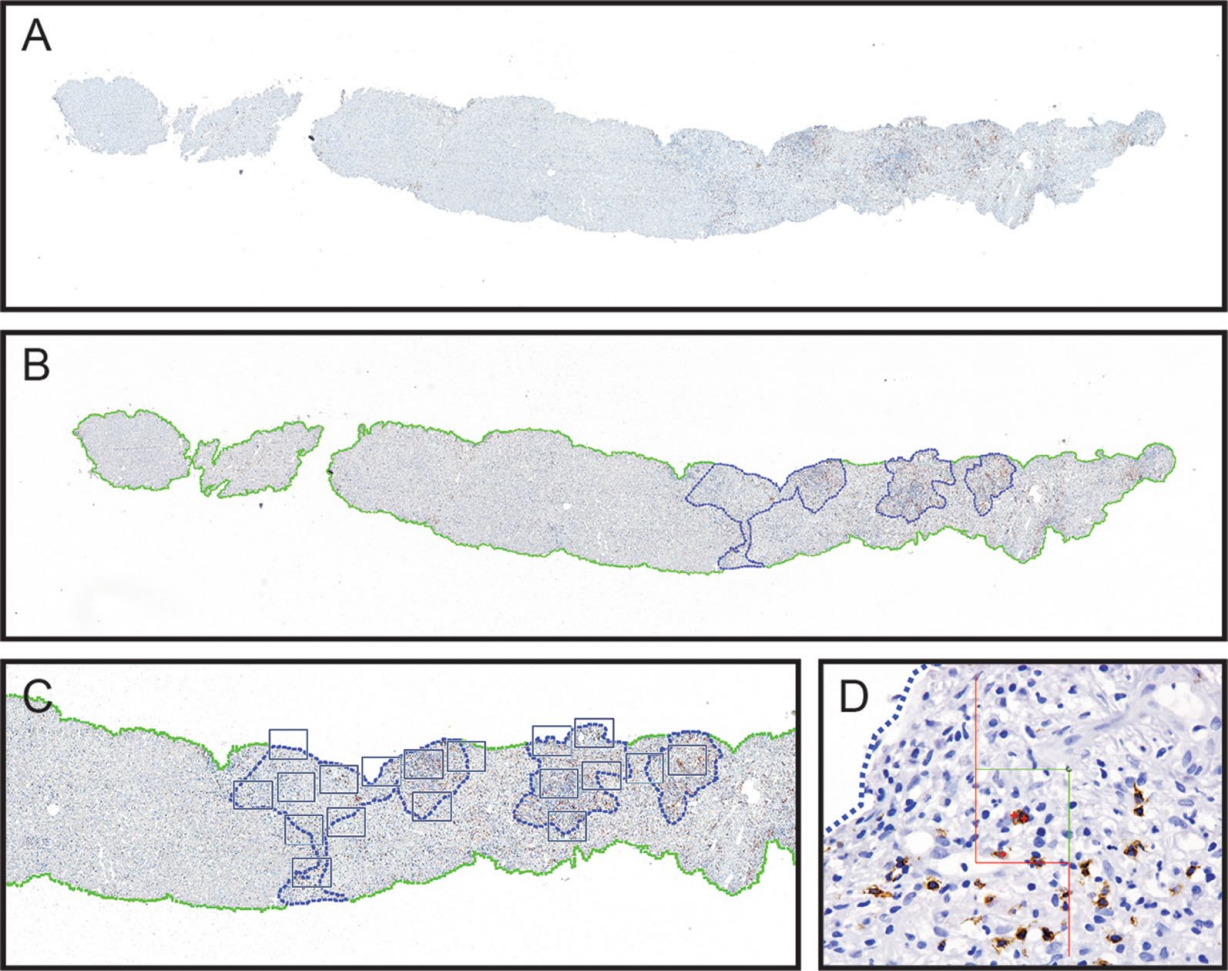
Representative images showing quantification procedure using NewCAST™ Visiopharm^®^ software. Super image capture of a tissue slide (**A**); computer-assisted drawing of the whole tissue (green line) and manually drawn portal areas as regions of interest (ROI) in blue (**B**); selected areas within the ROIs by random sampling (grey squares) (**C**); counting frame within one field of view at 400 magnification (**D**)

Initially, a super image of the whole slide was captured (Fig. 1A). Then, the tissue was automatically detected by the image software (green line). After that, the portal areas were manually outlined (blue dotted lines) and identified as the region of interest (ROI) (Fig. 1B, C). The computer assisted software, selected areas within these regions by systematic, random sampling (grey squares) (Fig. 1C). Following Visiopharm^®^ protocol and using the meander sample tool, cells in portal areas were counted. A counting frame of 30% within one field of view at 400 magnification (Fig. 1D) is accepted as an optimal percentage to achieve a statistically sufficient precision in the cell quantification (http://www.visiopharm.com). Higher sampling percentages are time-consuming and the same accuracy is achieved. All data were automatically recorded by the software and the results are shown in cells per mm^2^.

## Results

### Identification of cellular types found in the inflammatory infiltrates in biopsies from patients with dnIH

**Fig. 2:**
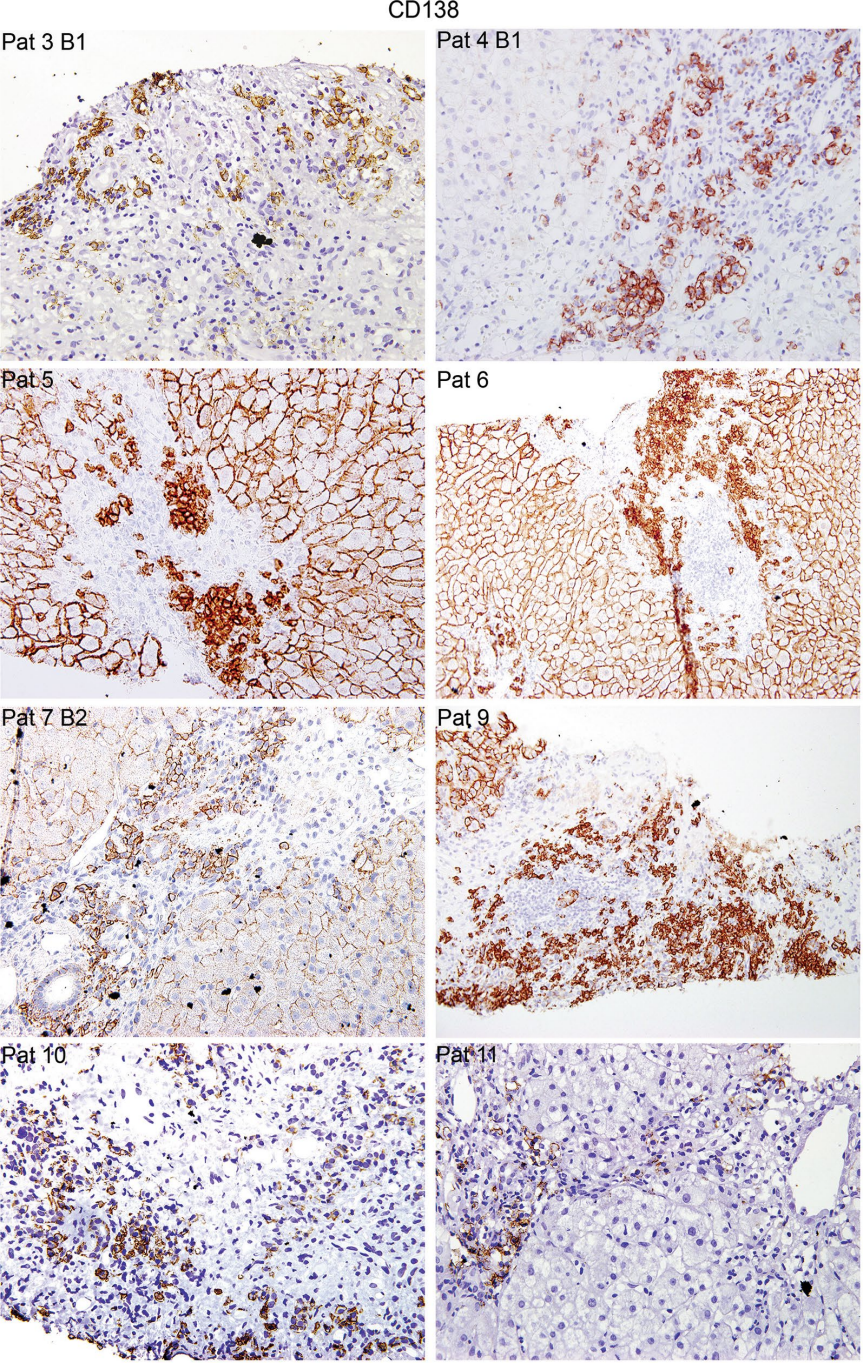
Representative images of dnIH diagnostic biopsies immunostained for CD138^+^ PCs, which are mainly found in the portal areas. Note that, in some biopsies, CD138 also stains the cellular membrane of hepatocytes. Biopsies from patients (Pat) 3, 4, 5, 7, 10 and 12 are shown at ×400 and Pat 6 and 9 at ×200 magnification

We analyzed the presence of CD138^+^ PCs mainly in portal areas, a characteristic feature of patients with dnIH (Fig. 2), and also screened for T cells, B cells, and macrophages in tissue samples obtained sequentially (Additional file 1: Figure S1). The tissue distribution of T lymphocytes was diffuse; T cells were mainly concentrated in the portal areas but also infiltrated a large number of sinusoidal capillaries (Additional file 2: Figure S2). B cells were significantly present in the infiltrates although to a lesser degree (Additional file 3: Figure S3). The macrophages also had a diffuse pattern, extended over most of the sinusoidal capillaries but also in the portal areas and around the central vein (Additional file 4: Figure S4). We also studied IgG4^+^ PCs, which were found in significant numbers in biopsies from patients with dnIH (Additional file 5: Figure S5). It was not possible to study all cellular markers in all the biopsies due to lack of availability of tissue samples. In total, we analyzed 12 diagnostic biopsies, 2 follow-up biopsies, and a prior-to-diagnosis biopsy informed as recurrence of the original virus C hepatitis.

### Cellular profile of the inflammatory infiltrates in the portal areas in patients with dnIH

**Fig. 3:**
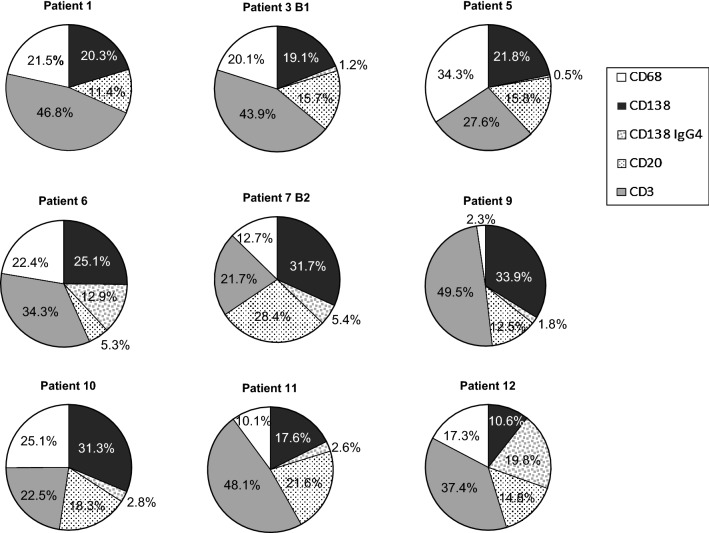
Relative contribution of immune cells in the inflammatory infiltrates in portal areas of liver tissue from 9 patients diagnosed with dnIH

In an attempt to define a cellular profile associated with this form of post-transplant pathology, we quantified the number of cells per mm^2^ of tissue (Table 2). We evaluated the relative presence of each cell type at the time of diagnosis in 9 biopsies for the 5 markers: CD138^+^ and IgG4^+^ (PCs), CD20^+^ (B cells), CD3^+^ (T cells), and CD68^+^ (macrophages) (Fig. 3). Considering that the biopsies were performed at very different post-transplant periods that ranged from 7 months to 7 years (see Table 1), the profiles were fairly similar. In general, there is an important contribution of CD138^+^ PCs (IgG4^−^ + IgG4^+^) with percentages that range from approximately 20% (patients 1, 3, 5 and 11) to 30% (patient 12) and even higher, between 34 and 38% (patients 6, 7, 9 and 10). IgG4^+^ PCs values are more variable, ranging from 19.8% of the total infiltrate in patient 12 to almost nothing (patients 1 and 5). PCs (IgG4^+^ + IgG4^−^) accounted for more than 20% of the entire infiltrate confirming the main feature of this type of rejection. The amounts of CD3^+^ T cells were variable, ranging from 21.7% (patient 7) to 49.5% (patient 9). The proportions of CD68^+^ macrophages were more similar, with 5 patients in the range of 20%, although there were some differences, with levels as high as 34.3% (patient 5) and as low as 2.3% (patient 9). The B cells were in general lower than PCs except for patient 11, who had a similar proportion of B cells and PCs. Although we could not study CD4 or CD8 markers in all the biopsies, we obtained the numbers in two samples and the proportion within the CD3^+^ population was 35.5% CD4^+^ versus 64.5% CD8^+^ cells for patient 5 and 19% CD4^+^ versus 81% CD8^+^ cells for patient 6.

**Table 2:** Inflammatory cell count in biopsies of 12 patients with de novo immune hepatitis

Patient	CD138^+^ IgG4^−^	CD138^+^ IgG4^+^	CD138^+^	CD20^+^	CD3^+^	CD68^+^	Total
Diagnostic biopsies							
1	–	–	600	333.3	1381.8	635.3	2950.4
2	–	–	–	375.7	1808.7	–	
3 B1	808.4	52.2	860.6	668.4	1860.9	852.3	4241.6
4 B1	–	–	1439.2	–	–	–	
5	1595	40	1635	1157	2019.6	2507.3	7318.9
6	1553.5	797.9	2351.4	325.2	2116.6	1381.5	6174.7
7 B2	935.9	160	1095.9	837.5	641.2	374	2948.6
8	–	–	–	244,1	2051.2	–	
9	1658.9	88.3	1747.3	613.5	2422.7	114.9	4898.9
10	899	81.3	980.3	524.5	645.2	721.2	2871.3
11	599.8	89.5	689.4	735.6	1636.6	344.3	3405.9
12	286.7	533.3	820	400	1008.7	466.7	2695.3
Follow-up biopsies							
3 B2	105.7	9.5	115.2	109.9	1296.1	630.8	215.2
4 B2	344.5	71.8	416.3	1591.2	3226.7	530	5764.2
Recurrent HCV prior to the development of dnIH							
7 B1	227.1	154.2	381.3	295.8	838.1	779.6	2294.8

Cell number is shown as cells/mm^2^

PC, plasma cell; B1, biopsy 1; B2, biopsy 2

### Cellular composition of the inflammatory infiltrates in biopsies from patients with chronic rejection

**Fig. 4:**
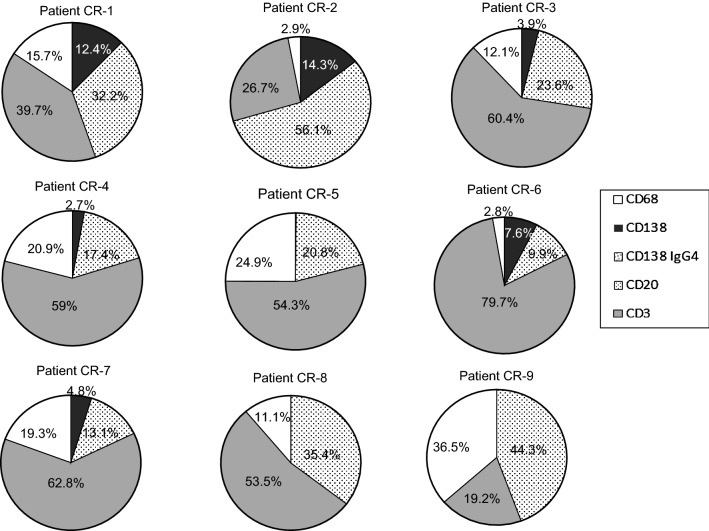
Relative contribution of immune cells in the inflammatory infiltrates in portal areas of liver tissue from 9 patients diagnosed with CR

We analyzed the same markers, except IgG4 due to the lack of tissue samples, in diagnostic biopsies obtained from a group of 9 patients with CR. The number of cells/mm^2^ tissue obtained after immunohistochemistry experiments is shown in Table 3. The results revealed that CD138^+^ PCs were practically absent in 3 of the 9 patients studied (CR-5, 8 and 9), present at a very low proportion in 3 patients (CR-3, 4 and 7) (2.7–4.8%), and present at higher values in the 3 remaining patients (CR-1, 2 and 6) (7.6–14.3%), but never reaching levels seen in the dnIH diagnostic biopsies. T lymphocytes were the most represented cell type in 7 of the 9 biopsies, with percentages higher than 50% in 6 patients and between 19.2 and 39.7% in the other 3 patients. The infiltrates also contained abundant B cells and macrophages (Fig. 4).

**Table 3:** Inflammatory cell count in diagnostic biopsies of 9 patients with CR

Patient	CD138^+^	CD20^+^	CD3^+^	CD68^+^	Total
CR-1	587.9	1529.4	1884.9	743.7	4745.9
CR-2	158.7	624	297.1	32.3	1112.2
CR-3	54.9	333.3	853.3	171.4	1413
CR-4	38.9	244.6	831.4	294.6	1409.5
CR-5	5.8	864.2	2250.7	1036.3	4157
CR-6	92.3	120	966.7	33.3	1212.3
CR-7	45.4	124.4	598.1	183.8	951.7
CR-8	11.6	1688.9	2552.2	531	4783.7
CR-9	4.6	676.9	292.6	556.5	1530.7

Cell number is shown as cells/mm^2^

CR, chronic rejection

### DnIH versus CR profiles

**Fig. 5:**
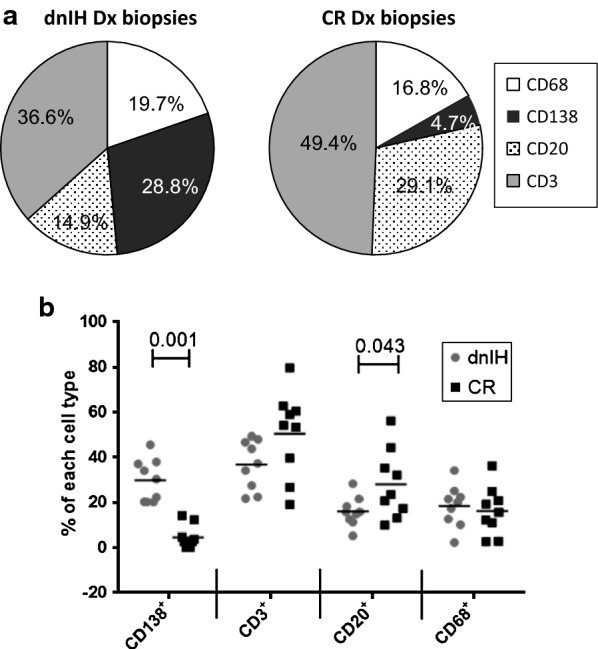
Comparison of the immune cell composition of the dnIH and CR groups. **a** Comparison of the mean values between the two groups. **b** Dot plot showing the statistically significant differences between cell markers in the dnIH and CR groups. Dx, diagnostic

By comparison of the cellular composition of biopsies from patients with dnIH versus CR, we propose a profile model for diagnosis of dnIH in liver tissue. The most distinctive feature of dnIH is the proportion of PCs, with a mean value of 28.8% that includes IgG4^+^ (4.9%) and IgG4^−^ (23.9%) compared with a mean value of 4.7% for the CR group. T cells predominate in the cellular infiltrates in both groups, with a mean of 36.6% in the dnIH group versus 49.4% in the CR group. Interestingly, B cells in CR biopsies were twofold the number in dnIH biopsies (29.1% versus 14.9%, respectively). The innate immune response represented by macrophages is very similar in both groups, with 19.7% in the dnIH group versus 16.8% in the CR group (Fig. 5a). Comparing the proportions of all four cell types studied, only PCs and CD20^+^ cells are statistically different between both groups (Fig. 5b). Not only the proportion but also the absolute numbers of CD138 cells/mm^2^ differentiate the two groups, with very low numbers found in CR biopsies, similar to those observed in pre- and post-treatment dnIH biopsies (Tables 2, 3). The nature of the immune response is clearly different and specific for each pathological process.

### The number of PCs in patients with dnIH decreases after steroid treatment

**Fig. 6:**
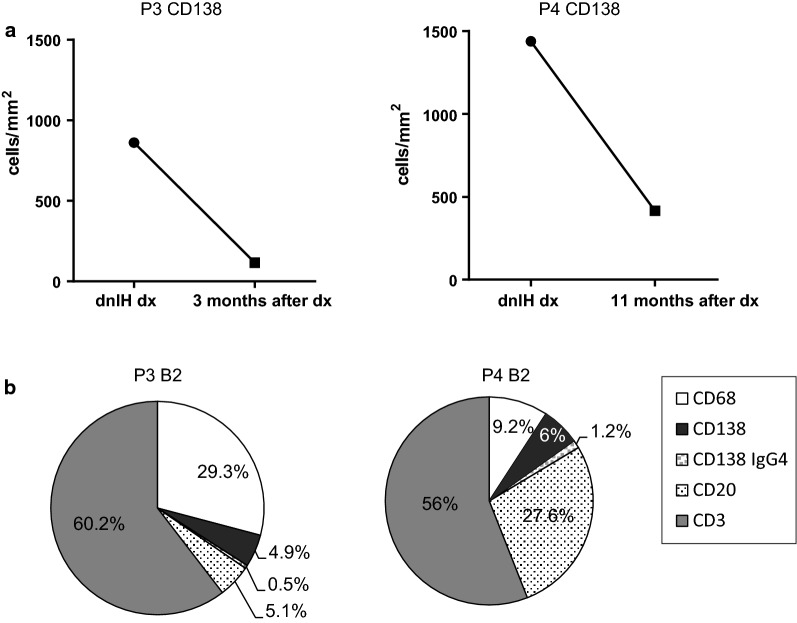
Cellular profiles after steroid treatment of dnIH patients 3 and 4. **a** CD138^+^ cell count of patient 3 at diagnosis and 3 months after treatment (left panel). CD138^+^ cell count of patient 4 at diagnosis and 11 months after treatment (right panel). **b** The cellular profile of patients 3 and 4 after treatment. rx, rejection; dx, diagnosis; B2, biopsy 2

We had the possibility to analyze 2 follow-up biopsies from patients 3 and 4, obtained 12 and 3 months after initiation of steroid treatment, the treatment of choice for dnIH in our hospital. We compared the total number of CD138^+^ cells in patient biopsies at the time of diagnosis and after steroid treatment. Both follow-up biopsies had an important reduction in the absolute number of PCs after treatment with steroids: from 860.6 to 115.2 in patient 3 and from 1439.2 to 416.3 in patient 4 (Fig. 6a). This reduction favored an increase in the relative abundance of CD3^+^ T cells in both follow-up biopsies that was higher than 50%, compared with the mean value in diagnostic biopsies of 36.6%. For CD20^+^ B lymphocytes, there was an evident reduction of B cells in the follow-up biopsy for patient 3 with 5.1%, far from the diagnostic value that was 15.7%, close to the mean value in dnIH diagnostic biopsies of 14.9%. Patient 4 had a high number of CD20^+^ cells (27.6%) although we do not have data on patient 4’s CD20^+^ cells at the time of diagnosis (Fig. 6b).

Macrophages were more abundant in patient 3’s follow-up biopsy, with 29.3% of the total cells, although not too different from the diagnostic biopsy, at 20.1%. In patient 4, the number of macrophages (9.2%) was lower compared with the mean diagnostic value (19.7%), although the proportion of CD68^+^ macrophages was the most variable among all diagnostic biopsies. The steroid treatment was effective in reducing the number of PCs, whose role in the pathogenic process seems to be relevant as confirmed by the patients’ improvement. The cellular profile after treatment was no longer a dnIH profile and was more similar to the profile of the CR group, especially with regards to PCs, for both patients.

### Cellular changes in the progression to dnIH

**Fig. 7:**
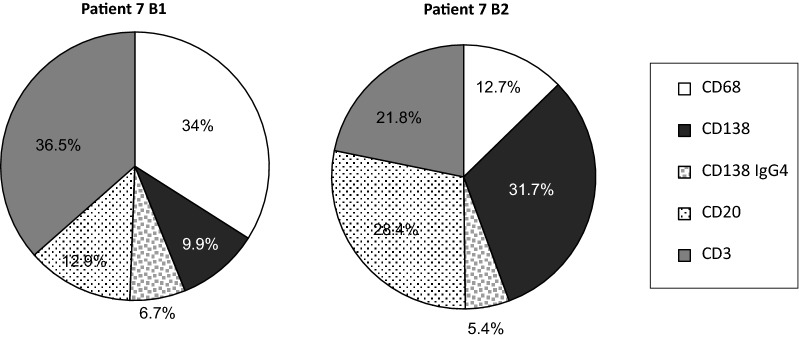
Cellular profiles of the inflammatory infiltrates in patient 7 recurrent HCV prior to development of dnIH (B1) and the diagnostic biopsy (B2)

The lack of protocol biopsies makes it very difficult to know the sequential changes in the inflammatory response leading to dnIH. Patient 7 presented with liver dysfunction 3 months after the transplant and the first biopsy (B1) showed histological features of hepatitis C recurrence and grade 1 acute rejection. Five months later, a second biopsy (B2) was necessary because the patient did not show any improvement in liver function. Analysis of B2 led to a dnIH diagnosis and prednisone treatment was prescribed with normalization of the liver enzymes. When we retrospectively analyzed patient 7’s biopsies, we observed that PCs were already present in B1 (9.9 + 6.7 = 16.6%), although not at levels comparable to the general dnIH profile defined in this study. The progression of the immune response was rapid, with a drastic increase in the number of CD138^+^ cells reaching 37.1% (31.7 + 5.4) PCs in B2. Together with B cells, the humoral arm in B2 represents more than half of the cellular infiltrate, whereas macrophages and T cells were substantially reduced (Fig. 7).

## Discussion

We defined the cellular types and relative proportions of the inflammatory infiltrates in dnIH diagnostic biopsies and in CR biopsies. The number of each cell type varied from one biopsy to another but the proportions in the dnIH group were similar in all the biopsies analyzed and extremely distinctive from the CR diagnostic biopsies. As expected, the main feature was the high proportion of PCs close to 30% of the total portal inflammatory infiltrate. Within the PC population, IgG4^+^ cells represented 17%.

Our hypothesis contemplates that during surgery, intracellular antigens such as the GSTT1 protein are released and become visible to B cells. After binding and internalization, the role of B cells is critical not only because they enter the affinity maturation program and differentiate into memory B cells and plasmatic cells, but also in their role as antigen presenting cells. At the time of diagnosis, the proportion of PCs and CD20^+^ cells in patients with dnIH is very characteristic and supports the new definition of dnIH as antibody-mediated rejection [8]. These two parameters are statistically different from the CR group where the presence of PCs is very low or absent and the inflammatory infiltrates are basically formed by CD20^+^ and CD3^+^ lymphocytes, together with macrophages. Not much is known about the presence of PCs in other liver post-transplant pathologies. One study reported a group of patients with acute cellular rejection of liver allografts who showed percentages of PCs from 10% and up to 30% as the severity of acute rejection was higher [9]. Furthermore, Fiel et al. used a scoring system to determine the ratio of PCs to the rest of the inflammatory infiltrate and found the highest score of PCs in biopsies at the time of de novo AIH diagnosis, which decreased in follow-up biopsies [2].

Furthermore, anti-GSTT1 antibodies are always IgG1 or IgG4 in similar proportions [6], supporting the reported bias towards IgG4^+^ PCs [10, 11].

In children, the nature of the inflammatory infiltrate of post-transplant de novo AIH was investigated. Unfortunately, the authors studied T lymphocytes and natural killer cells but not PCs or B cells. They found Th1 polarization but this feature was common to all 3 groups studied regardless of the type of post-transplant pathology, de novo AIH, acute rejection, and AIH [12].

Quantification of the number of cells per area of tissue not only facilitates the comparison with other post-transplant rejection processes, but is also very useful to control the progression to disease or response to treatment by observing changes in the cellular composition in follow-up biopsies. In this study, post-treatment biopsies showed not only a significant reduction in PCs but also in the total number of cells in the inflammatory infiltrates. This result is in accordance with those published by Pongpaibul et al. [13], who observed a reduction in the amount of PC-rich infiltrates in de novo AIH posttreatment biopsies.

In renal transplantation, high number of PCs have been confirmed in a subset of acute and chronic rejection processes acting as indicator of more adverse post-transplant outcomes [14]. In a different report, the number of PCs was found to be higher in CR than in acute rejection biopsies [15]. There seems to be a subset of PC-rich acute rejection distinct from the typical acute rejection, which is associated with poor graft survival [16, 17].

## Conclusions

In summary, this study reveals that there is a specific cellular profile in the portal inflammatory infiltrates associated with the occurrence of dnIH in the liver allograft, perfectly distinguishable from the CR profile, where the main differences reside on the proportion of PCs (sixfold higher) and of B lymphocytes (twofold lower). These findings are in accordance with a potential role for PCs in the pathology of dnIH and provide an extremely useful diagnostic tool as well as an additional histological marker to follow and evaluate a patient’s response to therapy. The fine characterization of the type and number of cells infiltrating the portal areas will contribute to the understanding of the mechanisms involved in this newly defined allograft rejection.

## Additional files

**Additional file 1: Figure S1.** Immunostaining of sequential slides of liver tissue of patient 3 B1.

**Additional file 2: Figure S2.** Representative images of dnIH diagnostic biopsies immunostained for CD3^+^ T lymphocytes. These cells are very abundant in the portal regions but are also disseminated in the capillary sinusoids of the entire tissue. All biopsies are shown at ×200 magnification with the exception of patient 6, at ×100 magnification.

**Additional file 3: Figure S3.** Representative images of dnIH diagnostic biopsies stained for CD20^+^ B cells. Biopsies from patients (Pat) 2, 6 and 7 shown at ×400 and Pat 3, 5 and 9 at ×200 magnification.

**Additional file 4: Figure S4.** Representative images of dnIH diagnostic biopsies stained for CD68^+^ macrophages. In some patients an accumulation of macrophages is observed in the portal areas, but in general they are in the capillary sinusoids distributed throughout the entire tissue. All biopsies are shown at ×200 magnification.

**Additional file 5: Figure S5.** Representative images of dnIH diagnostic biopsies stained for IgG4 plasma cells. All biopsies are shown at ×400, with the exception of patient 9, at ×200 magnification.

## Abbreviations

dnIH: de novo immune hepatitis
PC: plasma cell
CR: chronic rejection
AIH: autoimmune hepatitis
newCAST: new Computer-Assisted System Technology
ROI: regions of interest
