# Supplementary figures and images for: Identification of the cellular components involved in de novo immune hepatitis: a quantitative immunohistochemical analysis
Source: J Transl Med. 2018 Mar 13;16:62. doi: 10.1186/s12967-018-1440-8 (PMC5851325; doi:10.1186/s12967-018-1440-8)

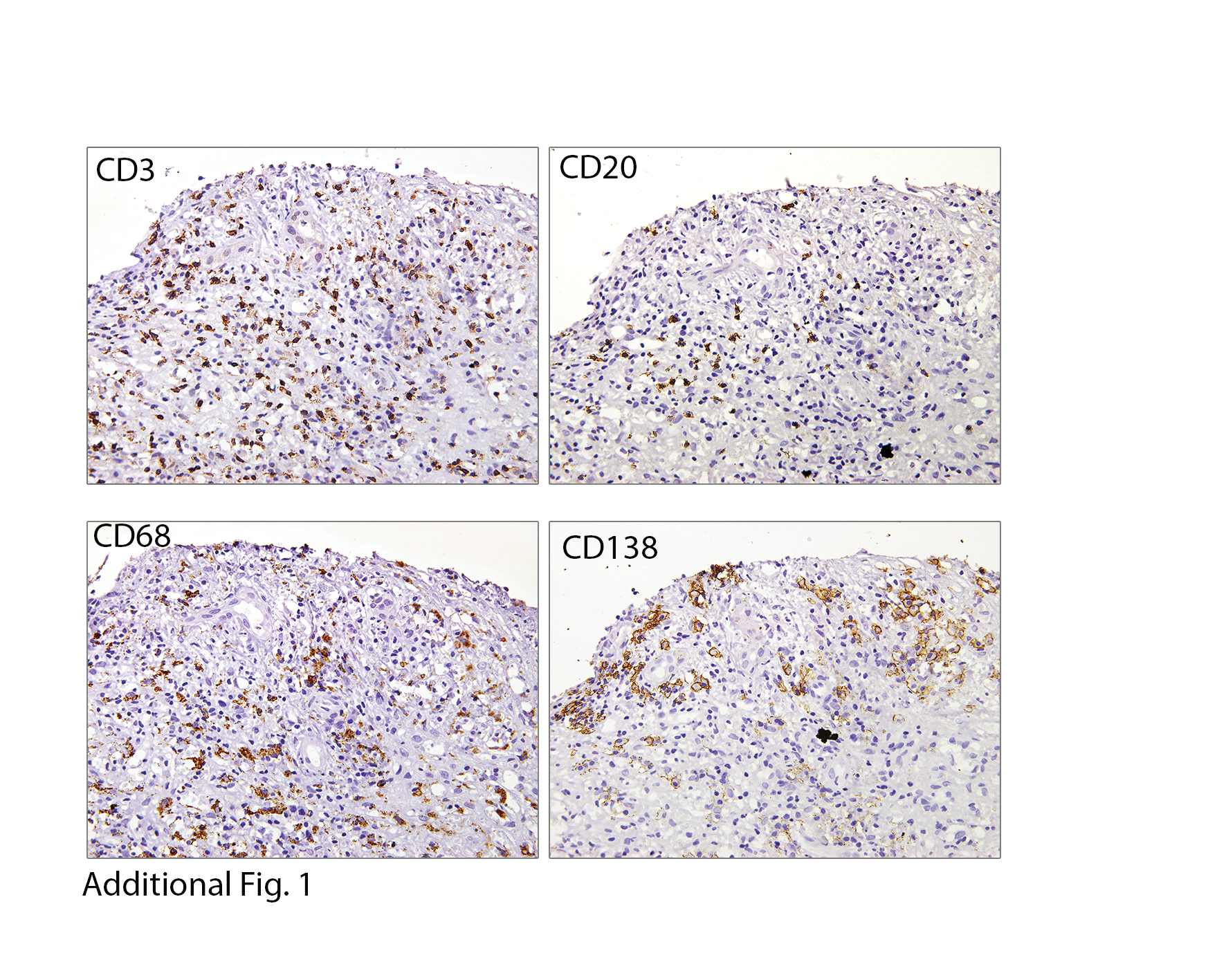

Supplement: Supplementary file 1 — Additional file 1: Figure S1. Immunostaining of sequential slides of liver tissue of patient 3 B1. [file 12967_2018_1440_MOESM1_ESM.jpg]

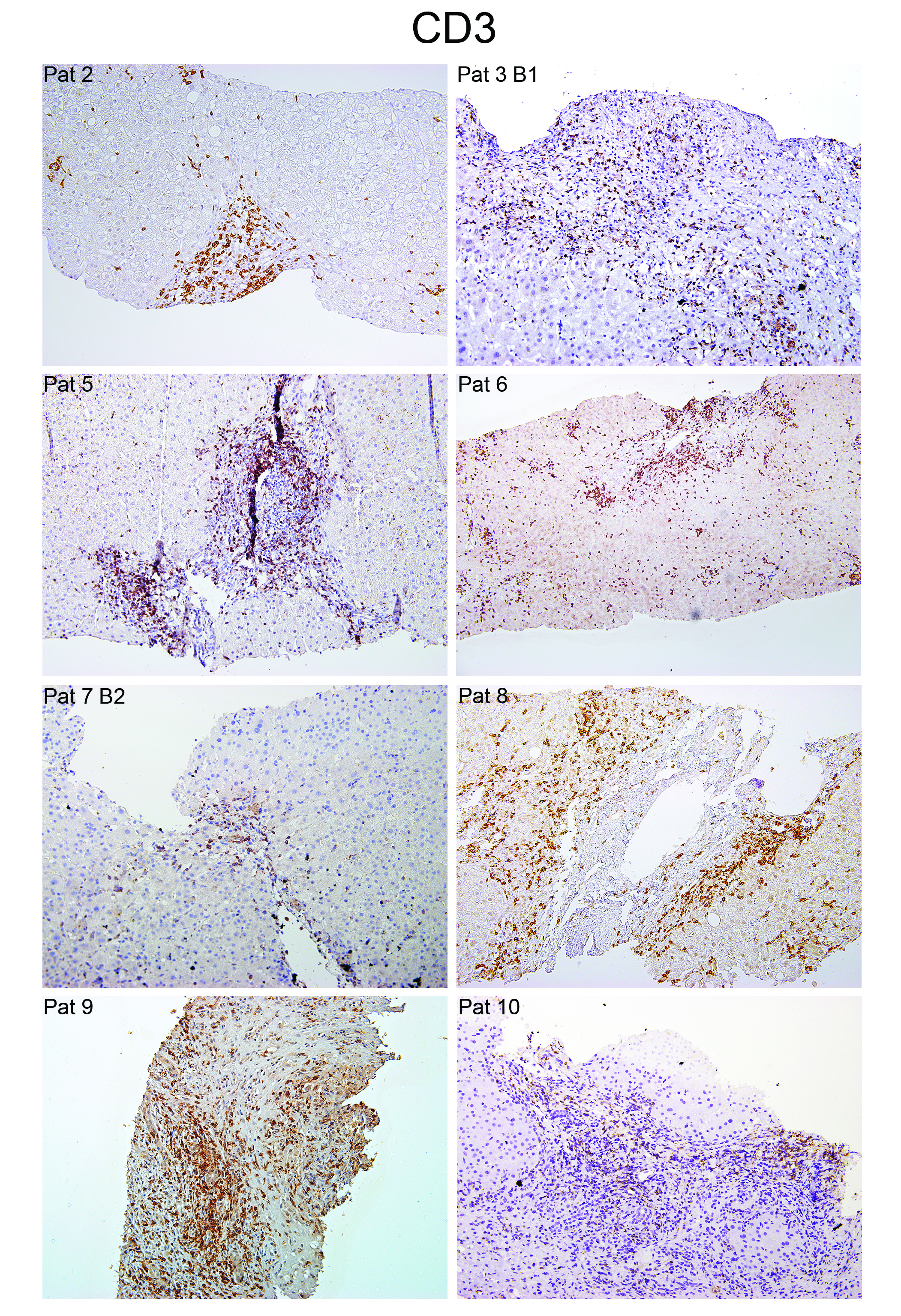

Supplement: Supplementary file 2 — Additional file 2: Figure S2. Representative images of dnIH diagnostic biopsies immunostained for CD3+ T lymphocytes. These cells are very abundant in the portal regions but are also disseminated in the capillary sinusoids of the entire tissue. All biopsies are shown at ×200 magnification with the exception of patient 6, at ×100 magnification. [file 12967_2018_1440_MOESM2_ESM.jpg]

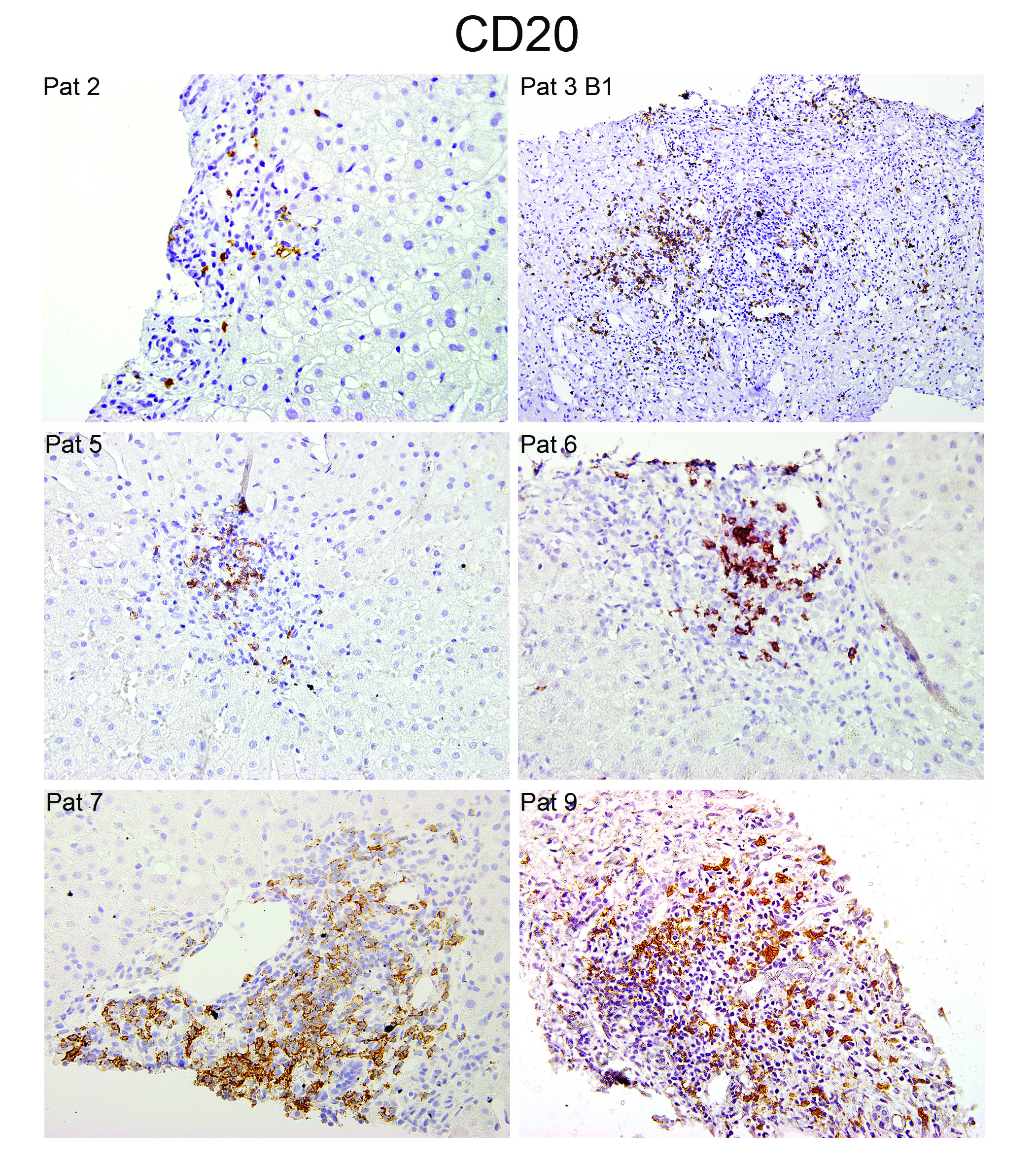

Supplement: Supplementary file 3 — Additional file 3: Figure S3. Representative images of dnIH diagnostic biopsies stained for CD20+ B cells. Biopsies from patients (Pat) 2, 6 and 7 shown at ×400 and Pat 3, 5 and 9 at ×200 magnification. [file 12967_2018_1440_MOESM3_ESM.jpg]

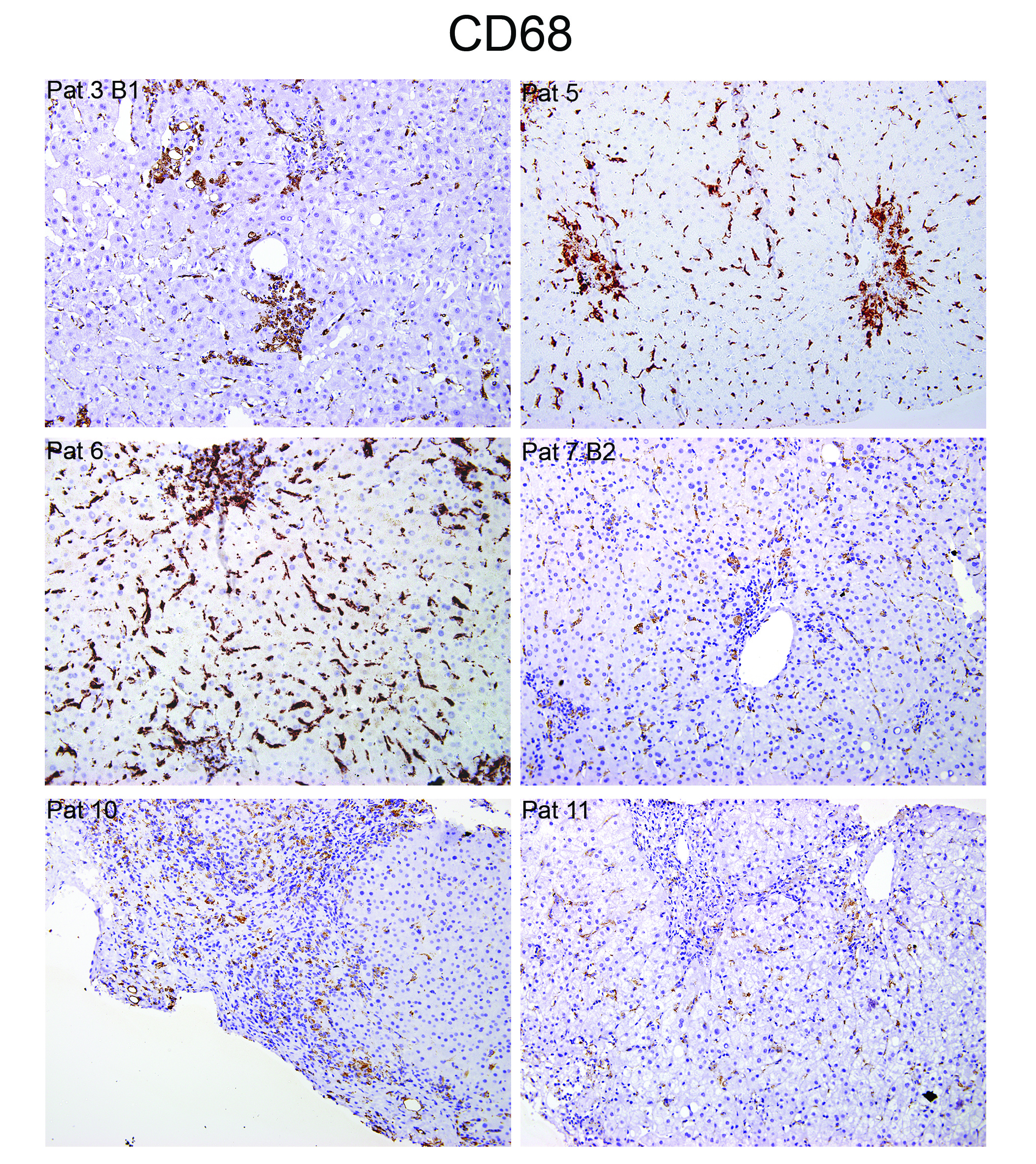

Supplement: Supplementary file 4 — Additional file 4: Figure S4. Representative images of dnIH diagnostic biopsies stained for CD68+ macrophages. In some patients an accumulation of macrophages is observed in the portal areas, but in general they are in the capillary sinusoids distributed throughout the entire tissue. All biopsies are shown at ×200 magnification. [file 12967_2018_1440_MOESM4_ESM.jpg]

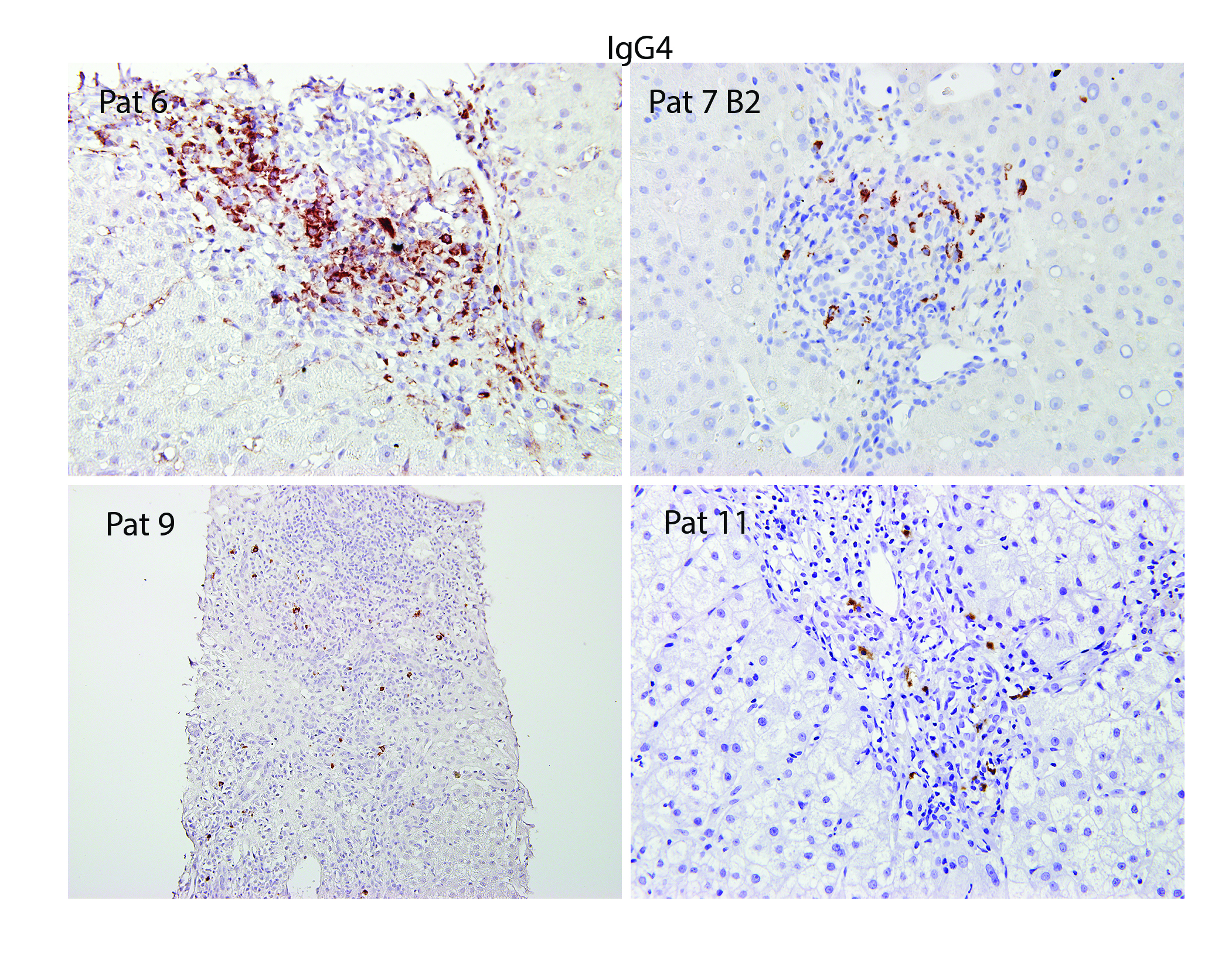

Supplement: Supplementary file 5 — Additional file 5: Figure S5. Representative images of dnIH diagnostic biopsies stained for IgG4 plasma cells. All biopsies are shown at ×400, with the exception of patient 9, at ×200 magnification. [file 12967_2018_1440_MOESM5_ESM.jpg]
